# Dynamic Changes of Brain Activity in Patients With Disorders of Consciousness During Recovery of Consciousness

**DOI:** 10.3389/fnins.2022.878203

**Published:** 2022-06-02

**Authors:** Yongkun Guo, Ruiqi Li, Rui Zhang, Chunying Liu, Lipeng Zhang, Dexiao Zhao, Qiao Shan, Xinjun Wang, Yuxia Hu

**Affiliations:** ^1^The Fifth Affiliated Hospital of Zhengzhou University, Zhengzhou, China; ^2^Henan Engineering Research Center for Prevention and Treatment of Brain Injury, Zhengzhou, China; ^3^School of Electrical Engineering, Zhengzhou University, Zhengzhou, China

**Keywords:** dynamics of brain activity, disorders of consciousness, high-definition transcranial direct-current stimulation, microstate, electroencephalogram (EEG)

## Abstract

The disorder of brain activity dynamics is one of the main characteristics leading to disorders of consciousness (DOC). However, few studies have explored whether the dynamics of brain activity can be modulated, and whether the dynamics of brain activity can help to evaluate the state of consciousness and the recovery progress of consciousness. In current study, 20 patients with minimally conscious state (MCS) and 13 patients with vegetative state (VS) were enrolled, and resting state electroencephalogram (EEG) data and the coma recovery scale-revised (CRS-R) scores were collected three times before and after high-definition transcranial direct current stimulation (HD-tDCS) treatment. The patients were divided into the improved group and the unimproved group according to whether the CRS-R scores were improved after the treatment, and the dynamic changes of resting state EEG microstate parameters during treatment were analyzed. The results showed the occurrence per second (OPS) of microstate D was significantly different between the MCS group and VS group, and it was positively correlated with the CRS-R before the treatment. After 2 weeks of the treatment, the OPS of microstate D improved significantly in the improved group. Meanwhile, the mean microstate duration (MMD), ratio of time coverage (Cov) of microstate C and the Cov of microstate D were significantly changed after the treatment. Compared with the microstates parameters before the treatment, the dynamic changes of parameters with significant difference in the improved group showed a consistent trend after the treatment. In contrast, the microstates parameters did not change significantly after the treatment in the unimproved group. The results suggest that the dynamics of EEG brain activity can be modulated by HD-tDCS, and the improvement in brain activity dynamics is closely related to the recovery of DOC, which is helpful to evaluate the level of DOC and the progress of recovery of consciousness.

## Introduction

Assessment of impairment and recovery of consciousness in patients with disorders of consciousness (DOC) is a challenge and major problem for clinicians. Thousands of patients with severe brain injuries lose their ability to communicate and fall into different comas [vegetative state (VS) and minimally conscious state (MCS)] every year. The recovery of consciousness of patients in different comatose states depends on the therapy after the evaluation of consciousness level. Clinical diagnosis and evaluation of patients’ conditions are mainly based on behavioral scales, such as the coma recovery scale-revised (CRS-R) (Giacino et al., 2014); however, there is a high misdiagnosis rate in the current scale scoring methods (Owen, 2019), which delays the subsequent treatment process of patients with DOC. Patients with MCS have greater potential for neurological recovery than patients with VS, and accurate evaluation is conducive to clinicians’ accurate and effective treatment of patients with DOC. Therefore, it is critical to study the different brain activity states of patients with MCS and VS, investigate the dynamic changes of brain activities during treatment, and accurately evaluate recovery of patients with DOC.

Several behavioral, neuroimaging, and electrophysiological methods have been used to assess the level of consciousness in patients with DOC, including the CRS-R (Xueliang et al., 2015; Annen et al., 2019), functional magnetic resonance imaging (fMRI) (Owen et al., 2006; Striano et al., 2010; Wu et al., 2015; Demertzi et al., 2019), positron emission tomography (PET) (Stender et al., 2014), electroencephalogram (EEG) (Goldfine et al., 2011; Faugeras et al., 2012; Sitt et al., 2014; Strauss et al., 2015; Gui et al., 2020; Wu et al., 2020), and multimodal imaging (Perri et al., 2016; Chennu et al., 2017). However, the CRS-R takes a long time and requires training of professional examiners, which is inconvenient for the assessment of patients with DOC, and fMRI is not appropriate to observe and track the dynamic recovery of consciousness due to its limitations such as high price, large size, inconvenient portability, and inability to conduct bedside clinical trials. EEG has the advantage of being portable and easy to deploy at the bedside, thus it is suitable to track the dynamic recovery of consciousness in patients with DOC. Silvia et al. used the perturbation complexity index (PCI) to grade patients with DOC based on the quantification of EEG responses to transcranial magnetic stimulation (Casarotto et al., 2016). This method can apply the cut-off value of independent validation of PCI to many patients with DOC to assess the sensitivity and complexity of patients with MCS and VS. However, the EEG at the selected time point was used to analyze patients with DOC during the PCI analysis, which only considered the static function of the brain, and cannot reflect the whole recovery process of consciousness in patients with DOC. Dehaene et al. believed that consciousness is characterized by a dynamic process of self-sustaining, coordinated brain activity that is constantly evolving, rather than a static brain function (Dehaene and Changeux, 2011). Correspondingly, some studies found that spontaneous recovery of consciousness from VS is accompanied by a functional restoration of the broad frontoparietal network and cortico-thalamo-cortical connections (Laureys et al., 2000; Aalderen-Smeets et al., 2006; Dehaene and Changeux, 2011). A previous study reported that dynamic changes in resting state activity during fMRI may provide specific cortical features for the loss of consciousness (Barttfeld et al., 2015). Similar to fMRI signals, we believe that the dynamic changes of EEG resting state activity can be also used to study the DOC. The dynamic changes of EEG resting state activity can be described by microstate, a global pattern of scalp potential topology, which changes dynamically over time in an organized manner (Lehmann et al., 1987). In other words, the functional state of the brain is a whole, and microstates analysis can detect different states of the brain, which provides a unique perspective for understanding the brain of patients with DOC.

The period of these quasi-stable field structures is called a microstate, which is considered to reflect the basic steps of brain information processing in spontaneous and event-related studies (Brandeis and Lehmann, 1989; Koenig et al., 1998). Four classes of standard EEG microstates maps have been repeatedly identified in numerous studies of healthy subjects of all ages (Koenig et al., 2002; Schlegel et al., 2012). Many related studies have demonstrated that the characteristics caused by dynamic changes in brain activity, such as personality types (Sverak et al., 2017) and behavioral states (Lehmann et al., 2010), are the drivers of changes in time series of EEG microstates. Therefore, the existence of specific microstates that reflect abnormalities in different brain activities and their inherent characteristics (including frequency and duration) can be regarded as quantifiable characteristic state markers for different neuropsychiatric and neurological diseases (Serrano et al., 2018). Additionally, other studies suggested that the dynamic brain activity, characterized by flexibility and temporal variability, is associated with cognitive function (Deco et al., 2011; Garrett et al., 2011; Schumacher et al., 2019). These EEG states are not meaningless repetitive patterns, but may indeed separate sensory and higher cognitive functions and correlate with levels of consciousness and task performance (e.g., the hierarchical level of language processing). EEG microstates are useful in probing the temporal dynamics of whole-brain neuronal networks. Some studies have investigated changes of EEG microstates in patients with neuropsychiatric diseases (Lehmann et al., 2005; Khanna et al., 2015). Indeed, Gui et al. used microstates in the process of evaluating the language processing of patients with DOC and combined the language stimulus paradigm to predict the recovery of patients with DOC, achieving good predictive classification results (Gui et al., 2020). Although there are reports in the literature that microstate D has been used to classify patients with MCS and VS (Stefan et al., 2018), microstate analysis has rarely been used to study DOC, and the relationship between the microstate and the dynamics of brain activity in patients with DOC is still unclear. Sverak et al. showed that repetitive transcranial magnetic stimulation over the left dorsolateral prefrontal cortex decreased the occurrence of microstate C in schizophrenic patients who responded positively to the treatment (Sverak et al., 2017). However, whether DOC’s dynamics of brain activity can be regulated is unknown. Our previous research has found that long-term high-definition transcranial direct current stimulation (HD-tDCS) promotes the recovery of consciousness in patients with MCS (Guo et al., 2019).

This study aimed to identify EEG microstate parameters with significant differences in patients with DOC after HD-tDCS treatment to track the dynamic recovery of consciousness. These parameters were used to assess the level of consciousness of patients with DOC to help to clinically distinguish VS from MCS and reduce the misdiagnosis rate. More importantly, specific information about dynamics of brain activity and recovery of consciousness is limited. Therefore, we combined long-term HD-tDCS and resting state microstates to explore the specific mechanism and relationship between recovery of consciousness and dynamics of brain activity in patients with DOC. Our results may be helpful to evaluate the level and recovery progress of consciousness in patients with DOC.

## Materials and Methods

### Participants

In this study, 35 patients’ EEG signals and CRS-R scores before and after HD-tDCS treatment were collected, and two patients were excluded because of insufficient the treatment course and poor EEG signal quality. Thirty-three hospitalized patients with chronic DOC (VS: three females and 10 males, 51.5 ± 8.7 years old; MCS: 10 females and 10 males, 52.3 ± 16.7 years old) were recruited in the Department of Neurosurgery at the Fifth Affiliated Hospital of Zhengzhou University (Table 1). The diagnosis of VS and MCS was based on five assessments within 10 days by DOC experts using CRS-R. Patients with DOC who had precuneus lesions, have had HD-tDCS treatment before and the duration of the consciousness disturbance less than 3 months were excluded. Patients who had pacemakers, aneurysm clips, other implanted devices, or other treatments/drugs that may have modified cortical-excitability were also excluded. This study was approved by the Ethics Committee of the Fifth Affiliated Hospital of Zhengzhou University (ethics number: KY2020024).

**TABLE 1:** Demographicand clinical data of the included patients.

DOC	Age	Sex	Etiology		CRS-R		
					T0	T1	T2
MCS (*n* = 20)	I (*n* = 14)	52	F	Hemorrhage	MCS(8)	MCS(9)	MCS (15)
		30	M	Hemorrhage	MCS(7)	MCS(10)	MCS (14)
		28	M	TBI	MCS(11)	MCS(13)	MCS (13)
		51	M	TBI	MCS(9)	MCS(10)	MCS (13)
		38	M	Hemorrhage	MCS(7)	MCS(10)	MCS(14)
		60	F	Hemorrhage	MCS(11)	MCS(13)	MCS(17)
		8	M	TBI	MCS(7)	MCS(11)	MCS(16)
		67	M	Hemorrhage	MCS(11)	MCS(11)	MCS(14)
		52	F	TBI	MCS(9)	MCS(10)	MCS(17)
		54	F	Hemorrhage	MCS(10)	MCS(11)	MCS(12)
		38	F	Hemorrhage	MCS(8)	MCS(10)	MCS(11)
		70	M	Hemorrhage	MCS(13)	MCS(16)	MCS(17)
		66	F	Hemorrhage	MCS(7)	MCS(10)	MCS(10)
		67	M	Hemorrhage	MCS(10)	MCS(11)	MCS(12)
	U (*n* = 6)	44	M	Hemorrhage	MCS(9)	MCS(10)	MCS(9)
		72	F	Hemorrhage	MCS(12)	MCS(13)	MCS (12)
		63	M	Hemorrhage	MCS(9)	MCS (9)	MCS (10)
		56	F	Hemorrhage	MCS(9)	MCS(10)	MCS (10)
		69	F	Hemorrhage	MCS(12)	MCS(13)	MCS (13)
		61	F	Hemorrhage	MCS(16)	MCS(17)	MCS (17)
VS (*n* = 13)	I (*n* = 4)	65	F	Hemorrhage	VS(6)	MCS (7)	MCS (8)
		51	M	Hemorrhage	VS(6)	MCS (7)	MCS (9)
		51	M	Hemorrhage	VS(6)	MCS (11)	MCS (13)
		62	F	Hemorrhage	VS(6)	MCS (10)	MCS (11)
	U (*n* = 9)	37	M	Hemorrhage	VS(6)	VS(6)	VS(7)
		48	M	Hemorrhage	VS(6)	VS(6)	VS(6)
		54	M	TBI	VS(2)	VS(2)	VS(3)
		40	F	Hemorrhage	VS(6)	VS(7)	VS(7)
		54	M	TBI	VS(6)	VS(6)	VS(7)
		66	M	TBI	VS(3)	VS(3)	VS(3)
		48	M	TBI	VS(6)	VS(6)	VS(7)
		41	M	Hemorrhage	VS(4)	VS(4)	VS(5)
		52	M	Hemorrhage	VS(2)	VS(2)	VS(3)

*DOC, Disorders of consciousness; MCS, Minimally conscious state; VS, Vegetative state; CRS-R, Coma recovery scale-revised; F, Female; M, Male; TBI, Traumatic brain injury; I, Improved group after HD-tDCS treatment; U, Unimproved group after treatment; T0, Before HD-tDCS treatment; T1, 1 week after HD-tDCS treatment; T2, 2 weeks after HD-tDCS treatment.*

### Method

#### Collection Method

Three periods of resting state data were collected, during which the subjects were in a naturally relaxed state. There was a 5-min interval between the two data collection sessions, and each session lasted for 3 min. EEG activities were recorded at 30 positions of the brain. The impedance of the channels was kept below 5 kΩ. Our previous studies found that HD-tDCS (Model 4x1-C2: Soterix Medical Inc, New York, NY, United States) over the precuneus could improve the recovery of consciousness and enhance information processing in the neural population of patients with MCS (Guo et al., 2019). All patients received HD-tDCS treatment (2 mA, 20 min, with an anode located above the precuneus) twice daily for 14 days. Detailed CRS-R evaluation was performed at three time points: before HD-tDCS treatment (T0), 1 week after HD-tDCS treatment (T1) and 2 weeks after HD-tDCS treatment (T2).

We divided patients with DOC into two groups based on the changes observed following treatment: unimproved group (Group U) and improved group (Group I). Group U: CRS-R increased less than 2 points and there was no new conscious behavior appeared; Group I: CRS-R increased more than 2 points, and new behavioral response of consciousness appeared.

#### Analysis Tools

Electroencephalogram signal preprocessing was completed by the EEGLAB toolbox in MATLAB software. Microstate analysis of EEG signals was performed by Microstate EEGLAB Toolbox (Technical University of Denmark) based on EEGLAB. Statistical analysis was performed by MATLAB software.

#### Electroencephalogram Signal Preprocessing

Electroencephalogram data preprocessing mainly consists of the following steps: (1) Referring to the previous microstate study (Koenig et al., 2002; Lehmann et al., 2005; Michel and Koenig, 2018), the finite impulse response (FIR) filter was used to conduct 2–20 Hz bandpass filtering on each channel to eliminate the influence of high-frequency noise since microstates are several different quasi-steady states in the alpha band (8–12 Hz) of the resting EEG signal. (2) Independent component analysis was used to remove electro-oculogram (EOG). (3) Bad channels and trials (body movement, muscle activity, and eye electrical signals not removed correctly) were manually removed. (4)The EEG data were re-referenced convert to common average reference.

#### Microstates Analysis

The preprocessed EEG signals were imported into the Microstate EEGLAB Toolbox to analyze the EEG microstates and calculate the parameters. We identified the point with the largest signal noise ratio (SNR) by calculating the global field power (GFP) at each time point in the time series. The calculation formula was as follows:


$$GFP=\frac{\sqrt{\sum_{i}^{k}{\left[V_{i}\left(t\right)-V_{mean}\left(t\right)\right]}^{2}}}{k}$$

where *V*_*i*_ (*t*) represents the instantaneous potential of the ith electrode at time *t, V*_*mean*_(*t*) represents the average instantaneous potential of all electrodes at time *t*, and *k* represents the number of electrodes (30 in this study). GFP represents the strength of the electric field on the brain at each moment, and is often used to measure the overall brain response to an event or to represent rapid changes in brain activity. The local maximum value of the GFP curve represents the moment of the strongest field intensity and the highest terrain SNR. In the resting state microstates analysis, it was found that the map at the peak of GFP was similar to the topographic map at surrounding time. The similarity between the trough and the surrounding map was low, indicating that the transition from one map to another may have been completed during a negative GFP peak. The electric field topography of the local maximum of the GFP curve is considered a discrete state, and the evolution of the signal is considered as a series of continuations and alternation of these states. Therefore, the peak time voltage amplitude of GFP was selected for cluster analysis. In this study, the modified K-means clustering algorithm was used for clustering.

The process of microstates analysis mainly includes the following three steps. Firstly, the GFP for each time point was calculated; at the maximum point of each GFP, we consider the spatial model of the EEG to be stable and account for most of the time series. Then, the improved K-means clustering method was used to cluster GFP. According to the existing research, the K-means clustering or improved K-means clustering method is used to determine the best number of clustering by cross validation (CV) criterion. We chose the number of clusters to be 4, because most studies found that 4 microstates prototypes are the most suitable for describing resting state EEG data, and clustering into 4 categories is convenient for us to compare with other studies. Then, patients in the MCS group and VS group before HD-tDCS treatment, patients in the improved group (Group I) before and after HD-tDCS treatment, and patients in the unimproved group (Group U) before and after HD-tDCS treatment were grouped into four groups of microstates. Finally, the original EEG signals of each subject were assigned to four microstates (A, B, C, and D) using the four microstates clustered by the patients as templates.

After the microstates analysis has been performed, the statistical properties of the microstates can be calculated, including the average GFP in all time frames of the same microstates class, the occurrences of each microstates class per second (OPS), the mean duration (MMD), each microstates class state class coverage (Cov), global explained variance and spatial correlation, and transition probabilities between the four microstates classes. We selected three types of microstates parameters: OPS, MMD, and Cov. The average GFP and the transition probability between microstates in the same microstate did not show valuable statistical results, so they were abandoned. The indicators derived from microstates dynamics are slightly different in neurophysiology. The MMD has been named the mean lifespan in some studies and has been interpreted to reflect the stability of its underlying neural components. The OPS may reflect the tendency of its underlying neural generator to be activated. The Cov quantifies the percentage of the total time that the microstates are dominant, which can be interpreted as reflecting the dominance of potential neurogenerators (Lehmann et al., 1987). Thus, a decrease in the microstates measure can be considered as a disengagement and instability of neural activity in the network that generates the microstates topographic map, and conversely, an increase may be a signal of neurological dysfunction.

### Statistical Analysis

Twenty patients with MCS and 13 VS patients who had been assessed and classified using the CRS-R scale were compared and analyzed before and after treatment. The CRS-R scores of 18 patients were improved after HD-tDCS treatment (MCS = 14, VS = 4) and 15 patients remained the same after treatment (MCS = 6, VS = 9). Three groups of microstate results were obtained:(1) Microstates of the VS group and MCS group before HD-tDCS treatment; (2) Microstates before and after HD-tDCS treatment in the unimproved group (Group U); (3) Microstates before and after HD-tDCS treatment in the improved group (Group I). For the three characteristic parameters of microstates MMD, OPS and Cov in each group, the two-tailed *t*-test was used to test the parameter differences between the groups, respectively. False Discovery Rate (FDR) correction was used to correct multiple two-tailed *t*-test results at three time points before and after treatment. Considering that although CRS-R has a high misdiagnosis rate, it is still the most commonly used method for clinicians to assess the recovery of consciousness in DOC patients. We conducted a Pearson correlation analysis between the obtained microstates parameters and their corresponding CRS-R, illustrating the reliability of the microstates analysis.

## Results

### Microstates of Patients With Vegetative State and Minimally Conscious State Before High-Definition Transcranial Direct Current Stimulation Treatment

**FIGURE 1:**
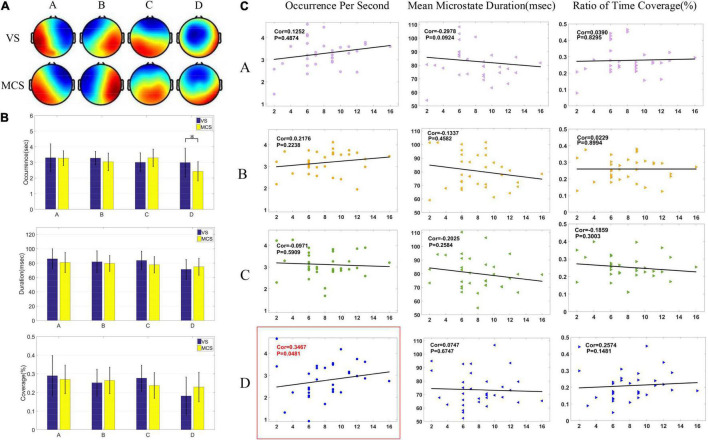
Microstates of patients with DOC before HD-tDCS treatment. **(A)** Microstate topographic map of patients in the VS group and MCS group before the treatment. **(B)** Comparison of three microstate parameters (OPS, MMD, and Cov) between the VS group and MCS group under four microstates by two-tailed *t*-test. There was a significant difference in OPS in microstate D between the two groups (*p* = 0.0425). **(C)** Pearson correlation between pretreatment microstate parameters and CRS-R scores in patients with DOC. Significant correlations (*p* < 0.05) were indicated in red and circled in red (Cor = 0.3467, *p* = 0.0481).

First, the resting state EEG microstates of the MCS group and VS group were analyzed before HD-tDCS treatment. The four microstates accounted for 69.28% of the generalized error variance (GEV) in the VS group and 74.01% in the MCS group. Microstates A and B of the two groups (as shown in Figure 1A) were consistent with the typical microstates A and B reported in the previous literature (Zhang et al., 2020). Microstate A is the right frontal-left posterior, microstate B is the left frontal-right posterior, microstate C is the middle frontal-occipital lobe, and microstate D is the middle frontal. Compared with the classic microstate topography in the previous literature, the four microstates topographies of DOC patients showed no significant difference in microstate A and B, but neither the MCS group nor the VS group matched standard microstate C and D. The standard microstate C shows an increasing trend from top to middle to the largest, which is a topographic map divided by a downward circular arc in the middle. The microstate C of the MCS group was relatively similar to the typical topographic map, but the increase is too fast to stop in the middle. The microstate C of VS Groups took on a different microstate C compared to typical topographic maps. The standard microstate D shows an increasing trend from the top to the middle to the largest, which was a topographic map divided by an upward circular arc in the middle. The MCS group was similar to the typical topographic map, but the increase was slow until it increased to below the middle. The VS group increased more slowly than the MCS group, and it increased to the bottom. Through the analysis of the topographic map, we believe that microstate C and D may be an important basis for distinguishing patients with DOC from healthy subjects. Two groups of patients who had been classified into VS and MCS after CRS-R evaluation before HD-tDCS treatment were divided into four groups by microstates analysis, as shown in Figure 1A.

Two groups of patients were subjected to two-tailed *t*-test to analyze the differences in microstates parameters. The differences among OPS, MMD, and Cov of the four microstates are shown in Figure 1B. As shown in Figure 1B, the MMD and Cov of microstate C in the MCS group were lower than the VS group, and the MMD and Cov of microstate D were higher than the VS group. Additionally, compared to the VS group, the MMD and Cov of microstate C showed a downward trend in the MCS group and the two parameters of microstate D showed an upward trend, but microstates A and B showed no obvious trend of change. Only the OPS of microstate D had relevant trends (*p* < 0.05).

After the microstates C and D were found to have a significant trend of change in the two groups of patients, correlation analysis was conducted with the three parameters of microstates C and D, as shown in Figure 1C. Figure 1C shows the Spearman correlation analysis results between CRS-R and three characteristic parameters of four microstates in 33 patients before HD-tDCS treatment. The OPS parameters of microstate D were positively correlated with CRS-R (*p* < 0.05), while other microstates parameters were not significantly correlated with CRS-R (*p* > 0.05).

### Dynamic Changes of Microstates Before and After High-Definition Transcranial Direct Current Stimulation Treatment in the Unimproved Group and Improved Group

**FIGURE 2:**
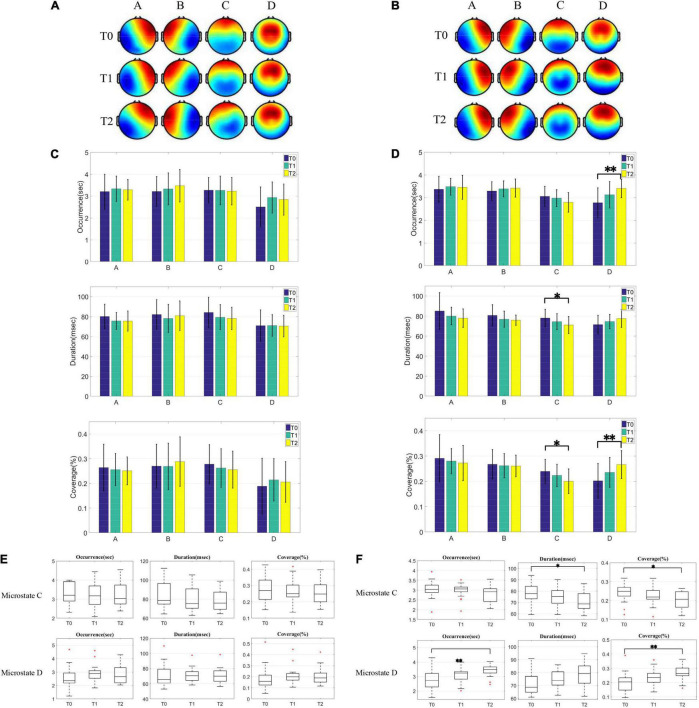
Comparison of microstates topographic maps and microstates parameters between the unimproved group and improved group. **(A,B)** Microstate topographic map of the unimproved group and improved group before and after HD-tDCS treatment. **(C,D)** Two-tailed *t*-test was used to compare the three microstate parameters (OPS, MMD, and Cov) in the four microstates between group U and group I before and after HD-tDCS treatment, and FDR correction was used to correct for multiple comparison results. **(E,F)** Dynamic changes of parameters of microstate C and D parameters in the unimproved group and improved group during HD-tDCS treatment.

The unimproved group and improved group were clustered into four microstates topographic maps before and after treatment for microstates analysis, as shown in Figures 2A,B. Compared with the classic microstates topography in the previous literature, the four microstates topographic maps of patients in group U showed no significant difference in microstate A, B, and C before and after HD-tDCS treatment, but microstate D did not match standard microstate D before and after the treatment. The microstate D of group U showed an increasing trend from top to middle and then to bottom before and after the treatment. Compared with the classic microstates topography, the four microstates topographic maps of patients in group I before and after the treatment showed no significant difference between microstate A and B, but microstate C gradually deviates from the standard microstate C after the treatment, increasing too fast from top to bottom. The microstate D of group I showed a trend of increasing from top to bottom before treatment, and it could be observed that the process of increasing microstate D gradually slowed down in the course of the treatment, showing a trend of increasing standard microstate D from top to middle. We can roughly observe that the microstate D tends to the standard microstate D after treatment in group U and group I.

We then calculated the microstates parameters of HD-tDCS before, 1 and 2 weeks after HD-tDCS treatment in the two groups, and performed *t*-test to analyze the recovery of patients in the two groups during the treatment. The *t*-test in Figure 2 is used to compare whether the microstates parameters of the same group at three time points before and after treatment are significantly different. We performed a two-tailed *t*-test on any two of these three time points, and adjusted the statistical test results using the FDR method in order to reduce the false-positive rate of multiple comparisons. We performed FDR correction on the three *P*-values of the three times comparison of any parameter, and the *P*-values shown in Figure 2 are all the results after FDR correction. It was found that no significant change of microstates parameters in the unimproved group during HD-tDCS treatment, as shown in Figure 2C. In contrast, *t*-test showed that MMD(*P* < 0.05) and Cov (*P* < 0.05) of microstate C were significantly different after 2 weeks of the treatment for the improved group, while OPS (*P* < 0.01) and Cov (*P* < 0.01) of microstate D were significantly different, as shown in Figure 2D. Additionally, during HD-tDCS treatment, the OPS, MMD, and Cov of microstate C showed a downward trend, while the three parameters of microstate D all showed an upward trend. These results were the same as the changes in microstates parameters found in the VS and MCS groups before HD-tDCS treatment. After HD-tDCS treatment, the improvement of scores were marked by the decrease of parameters of the microstate C and the increase of parameters of the microstate D, which was a dynamic process.

As shown in Figures 2E,F, we investigated the dynamic changes of microstates during HD-tDCS treatment. In Figure 2E, it was observed that the OPS, MMD, and Cov of microstate C showed a downward trend after HD-tDCS treatment, which were the same as the parameters changes trend of microstate C in the VS group and MCS group before HD-tDCS treatment. After 1 and 2 weeks of HD-tDCS treatment, the parameters of microstate D of the unimproved group were higher than before HD-tDCS treatment, but it did not increase gradually. It can be seen that the parameters of microstate C have a downward trend, while those of microstate D have an upward trend. However, no significant difference was found before and after HD-tDCS treatment, which corresponded to no improvement in CRS-R. In Figure 2F, after 1 and 2 weeks of HD-tDCS treatment, the parameters of microstate C in the improved group gradually decreased with the process of HD-tDCS treatment, while the parameters of microstate D gradually increased.

**FIGURE 3:**
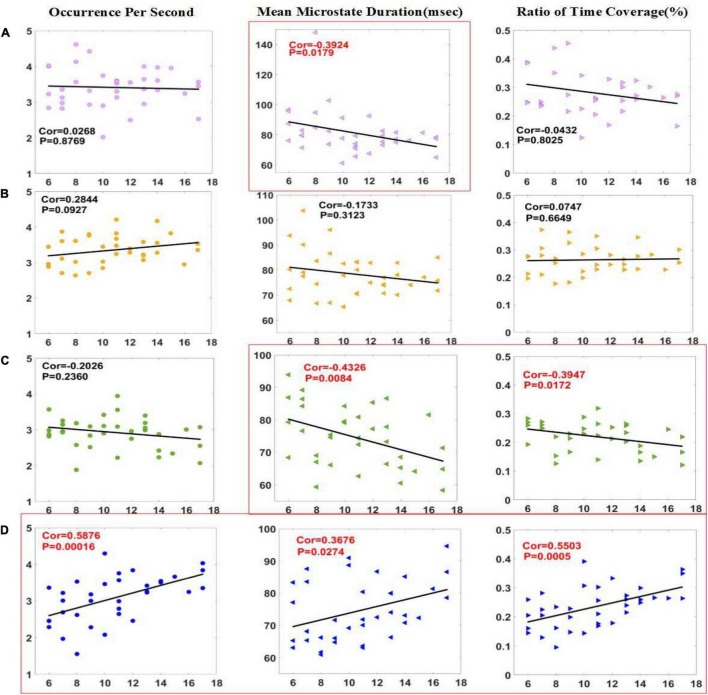
Correlation between parameters of microstate **(A–D)** and CRS-R scores in the improved group. Pearson correlation analysis was performed between the microstate parameters of patients with DOC and CRS-R scores in improved group. The MMD of microstate A and the MMD and Cov of microstate C were negatively correlated with CRS-R scores, and the three parameters of microstate D were positively correlated with CRS-R scores. Significant correlations (*p* < 0.05) were indicated in red and circled in red.

In Figure 3, correlation analysis was made between three microstates parameters and CRS-R scores of patients in the improved group before and 2 weeks after HD-tDCS treatment. The results showed that the MMD (*P* < 0.05) of microstate A, and the OPS (*P* < 0.001), MMD (*P* < 0.05), and Cov (*P* < 0.001) of microstate D were positively correlated with CRS-R. The four parameters with significant differences in the *t*-test are all in the list of significant correlation with the CRS-R scores. The two parameters of OPS and Cov of microstate D have a significant difference of less than 0.01 in the *t*-test and a correlation of less than 0.001 with the CRS-R scores, indicating that these two parameters are crucial in the assessment of the state of consciousness.

## Discussion

In this study, EEG microstates analysis was used to study the dynamic changes of brain functional state in patients with MCS and VS before and after HD-tDCS treatment, so as to determine the brain topological structure and temporal characteristics of subsecond brain activity. Additionally, the relationship between EEG microstate parameters and CRS-R scores and the grade of disorders of consciousness was also investigated. We found that the parameters of microstate D and C can be used to distinguish MCS from VS. Furthermore, dynamics of brain activity can be modulated by HD-tDCS, especially microstates C and D, and it is correlated with the session and time of the treatment. The dynamic changes of microstate C and D parameters correspond to the recovery process of consciousness in patients with DOC.

### Differences in Microstates Topographic Maps

The spatial topography of microstates C and D in patients with DOC were variable, while there was no difference in the topography of the other two types of EEG microstates. With the improvement of the CRS-R scores, the microstate C decreased and the microstate D increased. Studies have shown that the MMD of microstate D in patients with schizophrenia is significantly shorter and the OPS is lower than the normal level, indicating patients with schizophrenia and patients with disorders of consciousness have similar abnormalities (Serrano et al., 2018). Additionally, microstates C and D showed opposite directions of change in stroke patients, with microstate C less frequently present in the left side of the lesion and microstate D less present in the right side of the lesion, whereas healthy controls were in between (Koenig et al., 1999). We found that the higher the CRS-R in patients with DOC, the higher the level of consciousness state, which corresponds to the decrease of the parameters of microstate C and the increase of the parameters of microstate D. This opposite behavior of the two states suggests that the balance of dynamic interactions between the microstates is functionally more relevant than the specific changes in the individual states. Therefore, we believe that parameter abnormalities of typical microstates C and D can be used as one of the auxiliary diagnostic criteria for patients with DOC.

### Characteristics of Microstates Time

Before and after HD-tDCS treatment, CRS-R score is positively correlated with OPS in microstate D in patients with DOC. The higher the level of consciousness in DOC patients, the higher the OPS of microstate D. This suggests that OPS of microstate D can reflect the cognitive ability of patients with DOC. Several studies have shown that the resting state network (RSN) assessed by fMRI is significantly associated with the time progression of all four EEG microstate (Lehmann et al., 2005; Britz et al., 2010). Microstate A is mainly caused by the changes in negative blood-oxygen-level dependence (BOLD) activation of the bilateral superior and middle temporal parietal cortex, which is closely related to auditory network or sensorimotor (Gschwind et al., 2016; Michel and Koenig, 2018). Microstate B exhibits significant correlations with BOLD changes in the striate and extrastriate cortex and the negative BOLD activation in the bilateral occipital cortex, which goes hand in hand with the visual system (Britz et al., 2010; Yuan et al., 2012; Michel and Koenig, 2018). Microstate Cis related to positive BOLD activation in the bilateral inferior frontal cortices, the dorsal anterior cingulate cortex, and the right insular area, which is intimately connected with saliency network (Gschwind et al., 2016; Michel and Koenig, 2018; Yu et al., 2021). Microstate D is associated with negative BOLD activation in the right lateral ventral and dorsal regions of the frontal cortex and the parietal cortex, closely related to attention network (Gschwind et al., 2016; Michel and Koenig, 2018). The decrease of OPS in microstate C is related to the impairment of cognitive fatigue, and the significant decrease in the static state activity in the network region is due to the low Cov of microstate C (Andreou et al., 2014). Therefore, we infer that the resting state activity of the bilateral subfrontal cortex dorsal anterior cingulate cortex in patients with DOC is reduced to a certain extent. our results indicated that microstate C reflect the cognitive level of patients with DOC. When the parameters of microstate C of patients with DOC reduced closely to the normal level, it indicated the improvement of the level of consciousness. Microstate D is intimately connected with the attention network, so we infer that there is a certain increase in resting state activity in the right ventral and dorsal regions of the frontal cortex and the parietal cortex in patients with DOC after HD-tDCS treatment (Gschwind et al., 2016; Michel and Koenig, 2018), and our results also indicate that microstate D reflects the cognitive level of patients with DOC. This is consistent with the results of some previous studies on schizophrenia (Strelets et al., 2003; Irisawa et al., 2006; Kikuchi et al., 2007; Lehmann and Michel, 2011; Nishida et al., 2013; Andreou et al., 2014; Tomescu et al., 2015; Demertzi et al., 2019). In addition, One study showed that the percentage of time which was spent in microstate D in the alpha frequency band was the best measure for classifying VS and MCS (Stefan et al., 2018), and we also found similar results in our study.

### Dynamic Changes of Microstates Parameters During the Recovery Process of Consciousness

Patients in the improved group underwent a recovery process of consciousness due to the improvement of their CRS-R scores or the changes in their state of consciousness after HD-tDCS treatment. The dynamic changes of microstate parameters with significant differences during this process can be used as a recovery biomarker of consciousness. We found that in the recovery process of consciousness of patients in the improved group, the MMD and Cov of microstate C decreased while the OPS of microstate D increased. It can be concluded that the dynamic changes of microstate C and microstate D during HD-tDCS treatment can be used to evaluate the recovery of consciousness in patients with DOC. Additionally, before and during the 2 weeks HD-tDCS treatment in the improved group, the recovery process of patients’ consciousness was accompanied by the gradual decrease of microstate C parameters and increase of microstate D parameters, which was a dynamic gradual process of recovery. We believe that the cognitive level of patients with DOC is more likely to improve when the parameters of microstate C and microstate D are more significantly different before and after HD-tDCS treatment.

### Correlation Between Microstate Parameters and Coma Recovery Scale-Revised Scores

In patients with VS and MCS before HD-tDCS treatment, the OPS of microstate D was positively correlated with CRS-R scores. Patients rose from VS to MCS when the OPS of microstate D was relatively increased. The MMD and Cov of microstate C were negatively correlated with CRS-R scores before and after 2 weeks of HD-tDCS treatment in group I, while the OPS, MMD, and Cov of microstate D were positively correlated with CRS-R scores. The decrease of MMD and Cov in microstate C and the increase of OPS, MMD, and Cov in microstate D are closely related to the improvement level of consciousness, which corresponds to the increase in CRS-R scores. All of these dynamic changes can be used to evaluate the progress of the level of consciousness in patients with DOC.

### Limitation

There are several limitations in current study. Firstly, the sample size is small, and the modulation process of the dynamics of brain activity in patients with DOC obtained from the samples in the article may not be suitable for all patients with DOC. Secondly, there was no long-term follow-up of these patients with DOC to document their changes in the level of consciousness. Thirdly, we did not perform a multimodal evaluation (e.g., functional MRI), which would allow to better understand the dynamics of brain activity in patients with DOC during HD-tDCS treatment. Therefore, in the following study, more subjects will be included and their recovery process of consciousness will be followed up for long-term and multimodal evaluation.

## Conclusion

Our study found that EEG microstates dynamics were related to brain activity dynamics in patients with DOC. We found different spatial microstates in the brains of patients with VS and MCS by combining long-term HD-tDCS treatment with resting state microstates. Microstate C parameters decreased and microstate D parameters increased during recovery of consciousness. With the recovery of consciousness, patients with MCS have more obvious parameters changes than patients with VS, and the change of microstates is a dynamic process. Our results indicate that the recovery process of consciousness in patients with DOC is a dynamic change of brain activity, and the dynamics of brain activity in patients with DOC can be modulated by HD-tDCS.

## Data Availability Statement

The raw data supporting the conclusions of this article will be made available by the authors, without undue reservation.

## Ethics Statement

The studies involving human participants were reviewed and approved by the Ethics Committee of the Fifth Affiliated Hospital of Zhengzhou University. The patients/participants provided their written informed consent to participate in this study.

## Author Contributions

XW and YH contributed to design of the study. YG and RL collected the data. RZ, CL, and LZ contributed to EEG signal preprocessing and microstate analysis. DZ and QS performed the statistical analysis. YG and RL wrote the first draft of the manuscript. All authors contributed to manuscript revision, read, and approved the submitted version.

## Conflict of Interest

The authors declare that the research was conducted in the absence of any commercial or financial relationships that could be construed as a potential conflict of interest.

## Publisher’s Note

All claims expressed in this article are solely those of the authors and do not necessarily represent those of their affiliated organizations, or those of the publisher, the editors and the reviewers. Any product that may be evaluated in this article, or claim that may be made by its manufacturer, is not guaranteed or endorsed by the publisher.
